# Identification and Comparative Genomic Analysis of Type VI Secretion Systems and Effectors in *Klebsiella pneumoniae*

**DOI:** 10.3389/fmicb.2022.853744

**Published:** 2022-05-12

**Authors:** Wanzhen Li, Xiaofen Liu, Waitang Tsui, An Xu, Dan Li, Xuefei Zhang, Pei Li, Xingchen Bian, Jing Zhang

**Affiliations:** ^1^Institute of Antibiotics, Huashan Hospital, Fudan University, Shanghai, China; ^2^Key Laboratory of Clinical Pharmacology of Antibiotics, Shanghai, China; ^3^National Health Commission and National Clinical Research Center for Aging and Medicine, Huashan Hospital, Fudan University, Shanghai, China; ^4^State Key Laboratory of Genetic Engineering, Collaborative Innovation Center of Genetics and Development, Department of Biochemistry, School of Life Sciences, Fudan University, Shanghai, China; ^5^Phase I Unit, Huashan Hospital, Fudan University, Shanghai, China

**Keywords:** *Klebsiella pneumoniae*, type VI secretion system, effectors, antibacterial, toxins

## Abstract

*Klebsiella pneumoniae* is a nosocomial opportunistic pathogen that can cause pneumonia, liver abscesses, and infections of the bloodstream. The resistance and pathogenicity of *K. pneumoniae* pose major challenges to clinical practice. However, the ecology and pathogenic mechanisms of *K. pneumoniae* have not been fully elucidated. Among these mechanisms, the secretion systems encoded by strains of the bacteria confer adaptive advantages depending on the niche occupied. The type VI secretion system (T6SS) is a multi-protein complex that delivers effector proteins to the extracellular environment or directly to eukaryotic or prokaryotic cells. T6SSs are widely distributed in Gram-negative bacteria and play an important role in bacterial virulence and the interactions between bacteria and other microorganisms or the environment. This study aimed to enhance the understanding of the characteristics of T6SSs in *K. pneumoniae* through an in-depth comparative genomic analysis of the T6SS in 241 sequenced strains of *K. pneumoniae*. We identified the T6SS loci, the synteny of the loci in different species, as well as the effectors and core T6SS-related genes in *K. pneumoniae*. The presence of a T6SS was a common occurrence in *K. pneumoniae*, and two T6SS clusters are the most prevalent. The variable region downstream of the gene *vgrG* usually encodes effector proteins. Conserved domain analysis indicated that the identified putative effectors in *K. pneumoniae* had the functions of lipase, ribonuclease, deoxyribonuclease, and polysaccharide hydrolase. However, some effectors did not contain predicted functional domains, and their specific functions have yet to be elucidated. This *in silico* study represents a detailed analysis of T6SS-associated genes in *K. pneumoniae* and provides a foundation for future studies on the mechanism(s) of T6SSs, especially effectors, which may generate new insights into pathogenicity and lead to the identification of proteins with novel antimicrobial properties.

## Introduction

*Klebsiella pneumoniae* (*K. pneumoniae*) is an important opportunistic pathogen contributing to nosocomial and antimicrobial-resistant infections. The increasing prevalence of infections caused by multidrug-resistant *K. pneumoniae* has emerged as a major clinical and public health threat, while serious organ and life-threatening infections caused by highly virulent *K. pneumoniae* have also emerged (Russo and Marr, 2019; Wyres et al., 2020). Both drug-resistant and highly virulent *K. pneumoniae* have brought major challenges to clinical treatment and stimulated interest in studying *K. pneumoniae*. However, knowledge of the genomics, ecology, and pathogenicity of *K. pneumoniae* is relatively limited. Recently, the type VI secretion system (T6SS) was identified as a virulence factor in *K. pneumoniae* (Martin and Bachman, 2018). Furthermore, *K. pneumoniae* was found to exploit the T6SS nano-weapon to destroy bacterial competitors and fungi (Storey et al., 2020). However, the limited studies and lack of information on the T6SS in *K. pneumoniae* necessitate further exploration to clarify the physiological metabolism and pathogenic information of this clinically important bacterial species.

**FIGURE 1:**
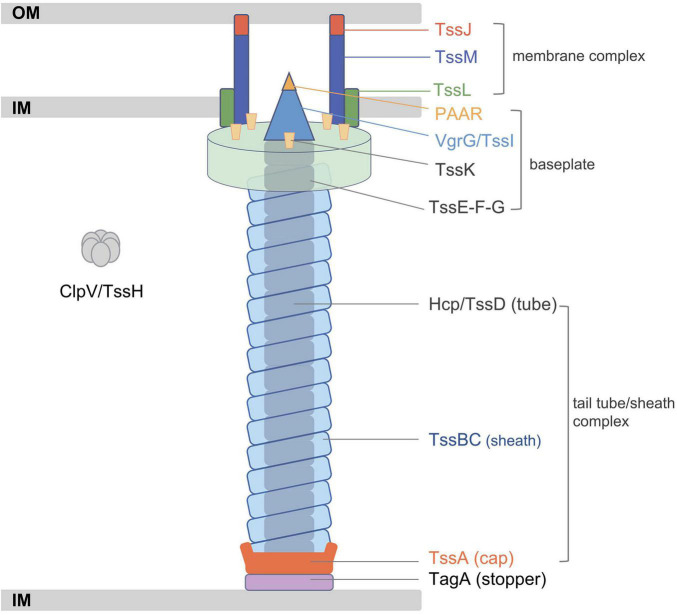
Schematic representation of the classic type VI secretion system (T6SS). Adapted from Cherrak et al. (2019). IM, inner membrane; OM, outer membrane.

The T6SS, which was first identified in *Vibrio cholerae*, is a multi-component apparatus that can deliver various effectors into eukaryotic or prokaryotic cells (Lien and Lai, 2017). T6SSs are widely distributed in Gram-negative bacteria and play a key role in the fitness of specific bacteria in different environmental niches (Wood et al., 2020). Although the composition of assembled proteins varies slightly among different bacteria species, the T6SS is usually composed of 13 core components named TssA–M (Cherrak et al., 2019). The T6SS is analogous to the T4 bacteriophage tail-like structure, containing a membrane complex, a baseplate, and a tail tube/sheath complex (Figure 1; Nguyen et al., 2018). The membrane-anchoring complex is typically composed of TssJ, TssL, and TssM, while the baseplate comprises at least six proteins. The contractile sheath of the T6SS is composed of two subunits, TssB and TssC, and the inner tube of the sheath is made of hemolysin-coregulated protein (Hcp, also termed TssD) topped by a spike complex of valine-glycine repeat G (VgrG, also termed TssI) and proline–alanine–alanine–arginine (PAAR) proteins (Ho et al., 2014; Wang et al., 2019). TssA localizes at the distal extremity of the TssB/C sheath and coordinates the polymerization of the structure, while TagA stops the assembly of the sheath and maintains it under the extended conformation. This assembly of the T6SS is a dynamic firing cycle. Components of the T6SS are recycled using energy provided by the ATPase ClpV (TssH) (Cianfanelli et al., 2016). In summary, bacteria utilize T6SSs to transport proteins into the environment and invade mammalian hosts or act as an antibacterial weapon by destroying surrounding competitors.

Effector proteins can be transferred by the T6SS machinery in two ways, namely, either through fusion with a structural component or by non-covalent interaction with one of the core components. In both cases, effectors are associated with the Hcp/VgrG/PAAR structure (Howard et al., 2021). The Hcp, VgrG, and PAAR proteins are the components of the T6SS, and in some bacteria, these proteins can also be extended by additional domains that may act as effectors (Pukatzki et al., 2007; Shneider et al., 2013; Ma et al., 2017a). The main function of T6SSs is inter-bacterial competition, and this phenomenon has been reported in *Acinetobacter baumannii*, *Pseudomonas aeruginosa* (*P. aeruginosa*), and other Gram-negative bacteria (Fitzsimons et al., 2018; Pissaridou et al., 2018). Other functions of the nanomachine T6SSs include survival against fungi, the transport of ions, and the targeting of eukaryotic cells (DeShazer, 2019). In addition, the discovery of effector proteins against eukaryotes has increased rapidly in recent years. Bacteria utilize T6SSs to participate in the physiological processes of eukaryotic cells, including adhesion, rearrangement of the skeleton, and evasion of host innate immunity to gain more advantages for colonization, spread, and survival (Monjaras Feria and Valvano, 2020). Two types of secretory phospholipase D (PLD) of *P. aeruginosa* have been identified as virulence effectors and can target both prokaryotic and eukaryotic host cells. These two PLD proteins trigger the killing of bacterial competitors and are internalized into non-phagocytes. PLDB also promotes intracellular invasion of host eukaryotic cells by activating the PI3K/Akt pathway (Bleves et al., 2014; Jiang et al., 2014). In addition, PAAR—one of the structural proteins of the T6SS—also plays a significant role in T6SS-mediated bacterial competition in bacteria, such as *Acinetobacter baylyi* and *P. aeruginosa* (Shneider et al., 2013; Tang et al., 2018). Most of the PAAR-type evolved effectors belong to the PAAR_like domain superfamily (cl21497). In general, different bacteria use and adapt their T6SS to perform specific roles depending on the host and environmental niches. Consequently, effector proteins are varied in biological and biochemical functions.

This study aimed to extend the present knowledge of T6SS clusters and effectors in *K. pneumoniae*. Analysis of the types and distributions of VgrG and PAAR within the species was also conducted as well as searching for possible evolved effectors. Different bioinformatic approaches were employed to analyze 241 strains of *K. pneumoniae* to further understand the characteristics of T6SS loci in this species.

## Materials and Methods

### Strains Used in This Study

A total of 27 clinical isolates of *K. pneumoniae* were kindly provided by the Institute of Antibiotics, Huashan Hospital. The genomic DNA of these strains was extracted using a Bacterial Genome DNA kit (Tiangen, China), and the whole genome of each isolate was sequenced using the Illumina NovaSeq 6000 platform. Quality checking and *de novo* assembly were conducted using the Fastx-toolkit and Velvet (version 1.2.03), respectively (Zerbino and Birney, 2008). Draft genome annotation was performed by searching against the National Center for Biotechnology Information (NCBI) non-redundant (nr) database. The complete genomes of all sequenced strains of *K. pneumoniae* available in the NCBI database (as of April 2021) were downloaded, and background information on each strain was collected from the BioSample database. Strains were then further screened to select those with complete background information, including geographic location, isolation source, host disease, and collection date. In addition, *K. pneumoniae* strains HS11286, NTUH-K2044, Kp52.145, MGH78578, and ATCC 43816 were included in the analysis because they contained a clearly identified T6SS in the literature (Sarris et al., 2011; Liu et al., 2012; Rodrigues et al., 2020). From the initial screening, a total of 214 strains were selected for analysis in the study. Detailed information on all strains used in the study can be found in Supplementary Table 1.

### Identification of T6SS Loci in *Klebsiella pneumoniae* Genomes

The integrated database SecReT6 (Li et al., 2015) was used to identify T6SS loci. All genome sequences were analyzed by BLASTp 2.10.1 + (*e*-value 1e-4) against experimentally validated T6SS clusters in the SecReT6 database. BLASPn (*e*-value 1e-5, > 70% nucleotide identity and > 70% coverage) was used to identify T6SS-related genes in the plasmids of the 214 strains with complete genome sequences. As 27 strains were draft genomes and plasmid sequence could not be determined, the identification of T6SS-related genes in the plasmids of these strains was not analyzed.

### Synteny Analysis

The type i2 T6SS clusters in different species were compared using Easyfig (version 2.2.5) with default parameters (e-value 1e-3). The nucleotide sequences and annotations of *Escherichia coli* 042 (NC_017626), *K. pneumoniae* HS11286 (NC_016845), *K. pneumoniae* (NZ_FO834906), *Enterobacter cloacae* ATCC 13047 (NC_014121), and *Burkholderia cenocepacia* H111 (NZ_HG938371) were downloaded from the NCBI RefSeq database.

### Prediction of T6SS Effectors

BLASTp (*e*-value 1e-5, > 50% amino acid identity) or RPS-BLAST was used to predict the potential effectors of *K. pneumoniae* T6SSs. Conserved protein domains were examined using the conserved domain database (CDD) at NCBI^1^ and AlphaFold2 (Jumper et al., 2021). Putative effectors were then classified based on their amino acid sequence similarity and conserved domains. Sequences of known T6SS effector (Tse) proteins were downloaded from SecReT6 (as of September 2021). In addition, PLD (CDO15120.1) effector protein was included in the analysis (Hsieh et al., 2019). The designed method would identify putative effector proteins with >50% amino acid coverage in the analyzed strains. In addition, the functional domains of proteins in the strains were considered to avoid missing identification; thus, if a domain of strain protein was consistent with the known effectors, it would also be identified. Domain visualizations were conducted with Tbtools (Chen et al., 2020).

### Phylogenetic Analysis

A homologous single-copy gene-based approach was employed to generate a phylogenetic tree of *K. pneumoniae* genome sequences. The STAG algorithm of OrthoFinder (version 2.5.4) was used to reconstruct a phylogenetic tree based on homologous genes (Emms and Kelly, 2019). Multiple sequence alignments of protein sequences, such as VgrG and PAAR, were performed using the Multiple Sequence Comparison by Log-Expectation (MUSCLE, version 3.8) algorithm (Edgar, 2004). The alignments were then refined using Gblock (Talavera and Castresana, 2007). FastTree was selected for the reconstruction of protein phylogenetic trees by the maximum-likelihood (ML) method. Tree visualization was performed using iTOL version 6^2^ (Letunic and Bork, 2021).

### Multilocus Sequence Typing and Annotation of Resistance and Virulence Genes

Multilocus sequence typing (MLST) of the strains was performed with mlst version 2.19.0 (Seemann T, mlst Github)^3^ (Jolley and Maiden, 2010). The Comprehensive Antibiotic Resistance Database (CARD) (Alcock et al., 2020) was used for the identification and annotation of antibiotic resistance genes, while the Virulence Factor Database (VFDB) (Chen et al., 2005) and BLASTp (e-value 1e-5) were used to identify virulence factors.

### Analysis of Proline–Alanine–Alanine–Arginine Proteins

Domain analysis of PAAR was conducted using the NCBI CDD^4^. The MEME online tool^5^ was applied for predicting the conserved motif structure of the PAAR family (Bailey et al., 2015).

### Analysis of Phospholipase D Effectors

Phyre2^6^ was utilized to predict protein structures similar to PLD superfamily protein in *K. pneumoniae* (Kelley et al., 2015). PLD three-dimensional structure prediction was conducted using AlphaFold2, and the predicted local distance difference test (plDDT) was used to evaluate the quality of the structure model (Jumper et al., 2021). The root-mean-square deviation (RMSD) calculated by Pymol was used to assess the similarity between protein structures (Delano, 2002). Alignments of the PLD protein sequences from *Arabidopsis thaliana* (NP_188194.1), *Homo sapiens* (AAH15033.1), *K. pneumoniae* (CDO15120.1), and *P. aeruginosa* PAO1 (AAG08474.1 and AAG06875.1) were performed using ClustalW multiple sequence alignment^7^ and colored with ESPript^8^ (Robert and Gouet, 2014). Domain analysis and visualization were performed using CDD and Tbtools, respectively.

## Results

### Widespread Distribution of T6SS Loci in *Klebsiella pneumoniae*

**FIGURE 2:**
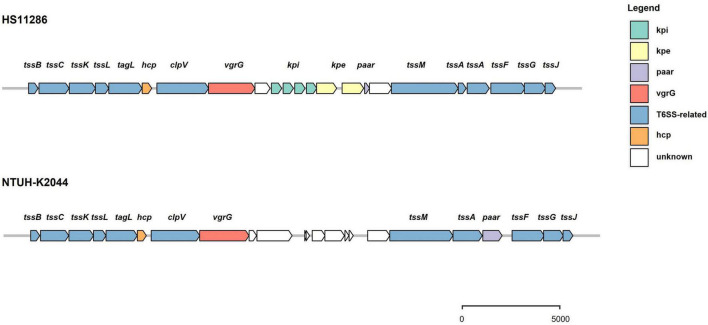
Schematic representation of intact T6SS loci in *K. pneumoniae*. There are two arrangements of gene structure. One is represented by strain HS11286 and features the *vgrG* gene followed by genes encoding several immunity proteins and their cognate effectors, and then the *paar* gene. Another form is represented by strain NTUH-K2044 and features the *vgrG* gene followed by unknown variable regions, the *tssM* and *tssA* genes, and then the *paar* gene.

The prevalence of T6SS loci was evaluated in 241 strains of *K. pneumonia*e, including strains available from the NCBI databases. Strains with background information (Supplementary Table 1) were included in the analysis. Genes encoding a T6SS cluster were present in all analyzed strains of *K. pneumonia*e. The majority of these isolates (229 of 241) harbor intact T6SS loci encoding conserved genes, and the *vgrG* gene is followed by variable regions that tend to have predictive immune-effector protein pairs (Figure 2). The T6SS gene cluster was incomplete in the remaining 12 strains. Further analysis showed that there were 1–4 T6SS loci in *K. pneumoniae*, and two clusters were the most common among the strains (178 of 241 strains), followed by three T6SS clusters (52 of 241 strains) (Supplementary Table 2). Numerous “orphan” genes encoding Hcp, VgrG, and PAAR were identified as being located outside the major T6SS gene clusters. This is congruent with those found in other species. Putative effectors are commonly encoded in the vicinity of these “orphan” genes (Huang et al., 2017; Ma et al., 2017a). There is no TagA protein encoded in the T6SS gene cluster of *K. pneumoniae*, but another protein, TagL, constitutes the T6SS protein complex and has been confirmed to be involved in the formation of the T6SS transmembrane complex in *E. coli* (Nguyen et al., 2021). The intact T6SS cluster in *K. pneumoniae* can be divided into two main types. One was represented by strain HS11286 (Figure 2), and the effector-immune protein genes appeared in clusters in variable regions, which was different from the effector-immune pairs in other bacteria, such as *Acinetobacter baumannii*. Another T6SS gene distribution pattern in *K. pneumoniae* is typical of strain NTUH-K2044, with no effector protein genes identified in the variable region and the location of PAAR differed from that of the first distribution type. In some strains, such as Kp52.145, PAAR proteins were not even identified. In addition, the T6SS cluster was not predicted in plasmids, but components of T6SS-related genes were identified (Supplementary Table 3).

### Synteny Analysis Among T6SS Loci in Five Strains of *Klebsiella pneumoniae*

**FIGURE 3:**
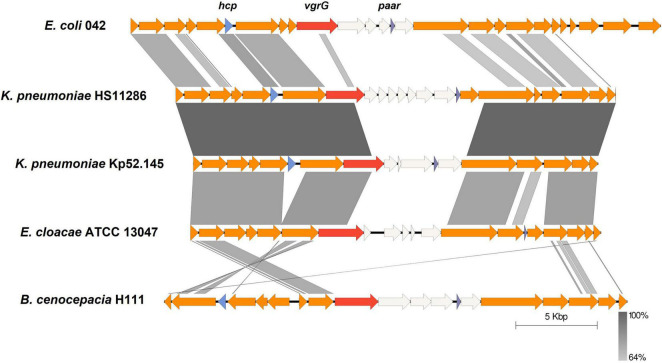
Comparative analysis of genome regions encoding T6SS of type i2. The genomes of different strains were aligned with BLASTn using Easyfig (e-value 1e-3). T6SS-related genes are represented by colored arrows and the key genes *hcp*, *vgrG*, and *paar* are labeled. Non-T6SS-related genes are white. Synteny regions between the sequences are displayed in gray blocks.

Employing the schemes proposed by SecReT6 and previous reports (Barret et al., 2011, 2013; Russell et al., 2014b), T6SS clusters are classified into i, ii, or iii types according to the structure of gene clusters and the evolutionary relationship of core components. The type i class contains six subtypes (i.e., i1, i2, i3, i4a, i4b, and i5), while type ii class is endemic to the *Francisella* pathogenicity island-encoded system. The type iii class was identified in Bacteroidetes. The complete T6SS cluster in *K. pneumoniae* belongs to the type i2 class according to the data in the SecReT6 database. In addition to the T6SS cluster in *K. pneumoniae*, the type i2 class of T6SS has also been identified in *E. coli*, *E. cloacae*, and *B. cenocepacia*. The T6SS gene cluster has previously been compared in *K. pneumoniae* (Sarris et al., 2011; Barbosa and Lery, 2019), and in this study, the type i2 T6SS clusters in strains of other bacteria were compared with those of *K. pneumoniae*. *K. pneumoniae* HS11286 and *K. pneumoniae* Kp52.145 were selected for comparison because they were strains with two and three T6SS gene clusters, respectively, as reported previously (Liu et al., 2017; Storey et al., 2020). Conservation of the type i2 class of T6SS gene cluster was analyzed in *E. coli* 042, *E. cloacae* ATCC 13047, *B. cenocepacia* H111, and the two previously mentioned strains of *K. pneumoniae*. There was high conservation of the T6SS-related genes within *K. pneumoniae* (Figure 3), and the type i2 T6SS gene cluster in *K. pneumoniae* was highly similar to that of *E. cloacae* ATCC 13047. However, the sequence and homology of the type i2 T6SS in *B. cenocepacia* H111 were quite distinct from those of *K. pneumoniae*. The middle variable regions in each strain were different and contained genes that encode unknown functional proteins. These results indicate the diversity of effector proteins. The key genes *hcp*, *vgrG*, and *paar* in the T6SS were depicted in different colors in all strains. The type i2 T6SS in the five strains all included *vgrG* and *paar* genes, but that of *E. cloacae* ATCC 13047 did not include *hcp* genes. The direction and sequence of some genes in the type i2 T6SS gene cluster of *B. cenocepacia* H111 were inconsistent with those of the other four strains. In addition, although some strains were identified as containing a type i2 T6SS cluster, they lacked some conservative T6SS genes, e.g., strain Kp52.145 did not have a *paar* gene. There were also strains with only one unknown type of T6SS identified, and the function of these T6SS clusters has yet to be elucidated.

### Classification and Characteristics of T6SS Effectors in *Klebsiella pneumoniae*

**FIGURE 4:**
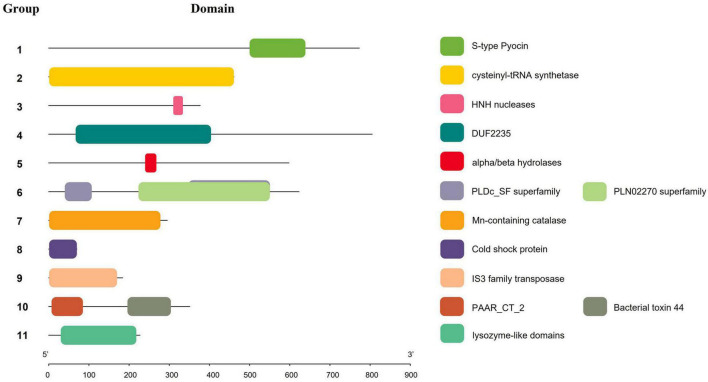
Schematic representation of domains of effectors in *K. pneumonia*e. Squares of different colors indicate different domains, and numbers represent effector protein groups.

The genomes of 241 strains of *K. pneumoniae*, which represented both historical and present isolates from a range of geographical locations, were analyzed, with the majority (75 of 241) belonging to ST11, followed by ST147 (18), ST15 (14), ST23 (12), and ST231 (12). To identify the putative effectors in *K. pneumoniae*, BLASTp and RPS-BLAST were performed. Given that Hcp and VgrG proteins are both components of T6SS cluster structure and effector proteins, and exist in each analyzed strain, they are not classified in Table 1. According to the classification methods in previous studies (Russell et al., 2014a; Li et al., 2015), effector proteins predicted in this study were classified into four types, namely T6SS DNase/RNase effector (Tde), T6SS lipase effector (Tle), T6SS effector (Tse), and T6SS peptidase effector (Tpe). The effector proteins were then divided into 11 groups based on their domains (the list of effectors for each strain is supplied in Supplementary Table 4), and a number was assigned to each group for identification. Table 1 and Figure 4 show the domains and functions of each group of putative effectors. Putative functions of effectors in *K. pneumoniae* included nuclease (group 1, 3, 10), lipase (group 6), Mn-containing catalase (group 7), and polysaccharides hydrolytic enzymes (group 11). Among them, effector proteins in group 1 were identified as S-type pyocin (pfam06958), which are polypeptide toxins produced by bacteria. S-type pyocins cause cell death through the breakdown of DNA due to endonuclease activity. Groups 4/5/6/10 were located in the downstream variable region of *vgrG* in the T6SS cluster. Group 4 contained a DUF2235 domain (pfam09994) whose specific function is unknown and usually found in clusters with multiple immune proteins. Group 5 effectors contained an alpha/beta hydrolase domain of unknown function. The effectors of group 6 were a class of lipase with a PLD superfamily domain (cl15239) that contains an H-x-K-x(4)-D signature motif. These PLD effectors were identified in no more than 16 strains and may be associated with the virulence of *K. pneumoniae* (Lery et al., 2014). The effector protein in group 10 was PAAR, a structural component of the T6SS, that contained the PAAR_CT_2 domain (cd14744) and the C-terminal toxin domain (pfam15607) with RNase activity. The group 10 effector proteins listed in this study refer to PAAR with a C-terminal toxic domain. Both groups 4 and 10 effector proteins were located in the intact T6SS cluster. Generally, if group 4 effector proteins were identified, PAAR was located in front of the *tssA* gene and contained only the PAAR_CT_2 domain. However, if the C-terminal toxin domain of PAAR was predicted, it was usually located downstream of *tssM*. The results predicted by Alphafold2 are shown in Supplementary Table 5, but the specific functions of most proteins in this study have yet to be elucidated.

**TABLE 1:** Putative effectors in *K. pneumoniae.*

Type ^a^	Identifier	Domains	Functions	Number ^b^
Tde	1	pfam06958, S-type Pyocin	endonuclease	28
	2	PRK00260, cysteinyl-tRNA synthetase	unknown	241
	3	cd00085, HNH nucleases	nuclease	1
Tle	4	pfam09994, DUF2235 domain-containing protein	unknown	132
	5	cl21494, alpha/beta hydrolases	unknown	23
	6	Cl15239, phospholipase D (PLD) superfamily	lipase	16
Tse	7	COG3546, Mn-containing catalase	hydrogen peroxide catalyst	217
	8	Cold shock protein	unknown	241
	9	IS3 family transposase	unknown	7
	10	pfam15607, Bacterial toxin 44	ribonuclease	30
Tpe	11	cl00222, lysozyme-like domains	polysaccharides hydrolase	1

*^a^Effectors classification is: Tle, T6SS lipase effector; Tde, T6SS DNase/RNase effector; Tse, T6SS effector; Tpe, T6SS Peptidase Effector, ^b^Indicates the number of genomes containing effectors identified in 241 K. pneumoniae strains used in this study.*

**FIGURE 5:**
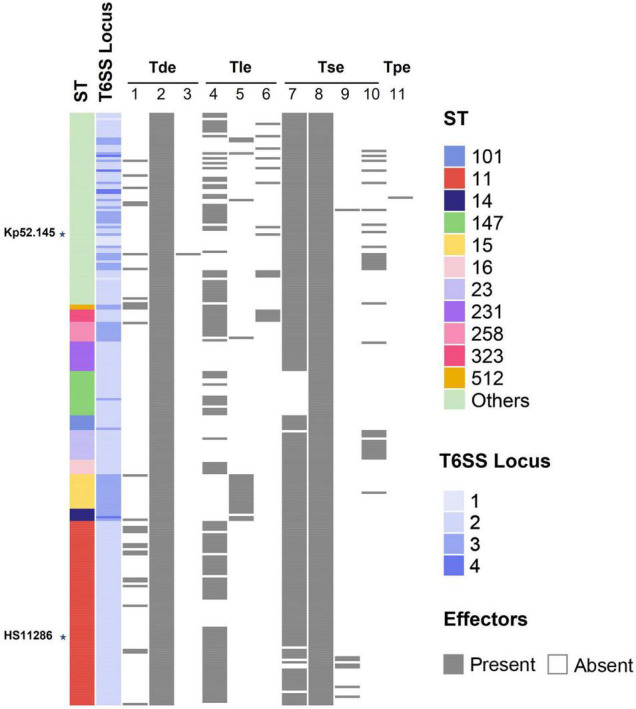
Schematic representation of the relationship between ST type and T6SS effectors. Each color represents an isolate of a different ST type. The number of T6SS clusters (1–4) is shown in a blue gradient. The presence and absence of effector proteins are indicated by black and white rectangles, respectively. The labeled stars indicate the listed strains.

**FIGURE 6:**
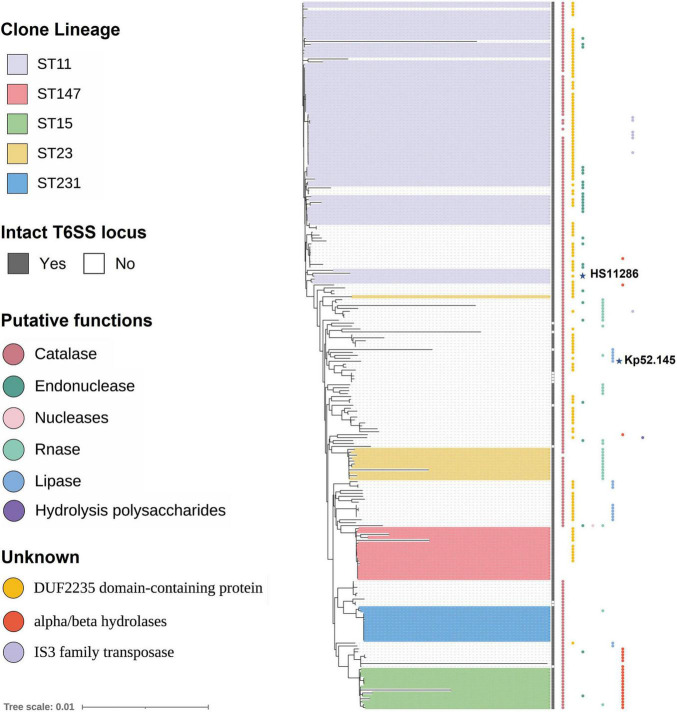
Phylogenetic trees of 241 strains of *K. pneumoniae* showing T6SS effectors. Strains enclosed by the purple box are ST11 strains. Similarly, different colored boxes show strains of different ST types. The predicted effectors encoded by each strain are indicated by the colored circles at the tip of strain name, with the color of each circle representing the predicted type of each effector. Refer to Supplementary Files 1 for raw phylogenetic tree data. The labeled stars indicate the listed strains.

A comparison of the background information of 241 strains of *K. pneumoniae* (Figure 5) revealed that all ST11 strains had two T6SS clusters and one of the clusters was intact. ST14 strains had three to four T6SS clusters, while all ST15 strains had three T6SS loci. However, due to the small number of ST14 and ST15 strains, the characteristics of the T6SS gene clusters require verification. For the distribution of effectors in isolates of different ST types, groups 1, 2, 4, 7, 8, and 9 effector proteins were identified in ST11 *K. pneumoniae*, and most ST11 isolates (60 of 75) had a group 4 effector protein with the DUF2235 domain. However, these effectors were not identified in ST14, ST15, and ST231 isolates. Group 5 effector proteins containing an alpha/beta hydrolases domain were predominantly distributed in ST14 and ST15 strains, while group 10 effector proteins (PAAR with a C-terminal toxin domain) were predominantly distributed in ST23 strains. In addition, all ST323 strains analyzed in this study contained group 6 proteins, which have predicted PLD activity. The corresponding relationship between ST type and effector is shown in Supplementary Table 2. Although the identified repertoire of potential effector proteins is diverse, almost all examined ST lineage strains shared identical predicted effectors regardless of the country of isolation or site of infection. To determine whether related strains of *K. pneumoniae* expressed similar groups of effectors, an ML phylogenetic tree of all strains included in these analyses was generated from a homologous single-copy gene (Figure 6). Functionally similar effectors were largely found in closely related strains, such as alpha/beta hydrolases (group 4). This result was consistent with the above findings.

### Diversity of VgrG Proteins Encoded in *Klebsiella pneumoniae*

**FIGURE 7:**
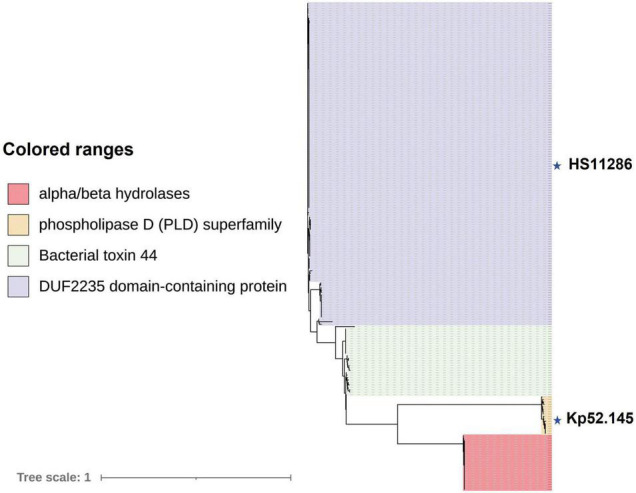
VgrG proteins of *K. pneumoniae* are separated into distinct phylogenetic classes. An ML phylogenetic tree was generated from 201 aligned VgrG protein sequences. Blocks of the same color indicate that those effector proteins encoded downstream of *vgrG* have similar functions. Effectors downstream of *vgrG* with an alpha/beta hydrolase domain are indicated in red, phospholipase D (PLD) superfamily, DUF2235 domain-containing protein, and bacterial toxin 44 effector proteins predicted downstream of *vgrG* are indicated in yellow, purple, and green, respectively. The labeled stars indicate the listed strains.

Effector proteins are usually predicted in variable regions downstream of *vgrG*. Analysis of the T6SS cluster of *K. pneumoniae* revealed that four Tses were located downstream of *vgrG*, and the domains of these effectors included DUF2235 (pfam09994), bacterial toxin 44 (pfam15607), PLD superfamily (such as cl15239), and alpha/beta hydrolases (pfam06958). One of the effectors was PAAR, which is also a component of the T6SS. The VgrG corresponding to the first two effectors mentioned above belongs to the complete T6SS gene cluster. To delineate the relationship(s) among the VgrG proteins, a total of 201 VgrG amino acid sequences corresponding to the above four effector proteins were analyzed by ML phylogenetic tree (Figure 7). This phylogenetic analysis revealed that closely related VgrG proteins were always found upstream of similar effector-encoding genes. The VgrG of 47 strains could predict both DUF2235 and PAAR with the C-terminal toxic domain. Most VgrG proteins contained the same VgrG domains, including the vgr_GE superfamily (cl36942) and T6SS_Vgr (pfam13296) domains, while the C-terminal region of each VgrG protein contained an amino acid region with unknown function (DUF2345). However, most of the VgrG proteins (28 of 29) corresponding to bacterial toxin 44 do not have T6SS_Vgr domains.

### Proline–Alanine–Alanine–Arginine Proteins Show a Toxic Domain in Some Strains of *Klebsiella pneumoniae*

**FIGURE 8:**
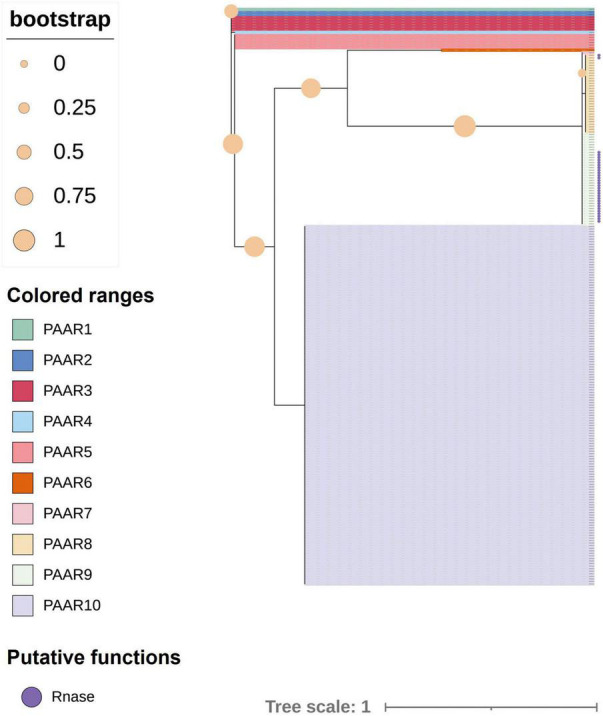
Phylogenetic tree of proline–alanine–alanine–arginine (PAAR) proteins in 226 strains of *K. pneumoniae*. The tree was generated from PAAR amino acid sequences of intact T6SS clusters using FastTree. Filled circles at nodes indicate bootstrap values. Groups 1–10 represent different PAAR proteins with different colors. Purple circles indicate PAAR proteins with a bacterial toxin 44 domain at the C-terminus.

The analysis of 241 *K. pneumoniae* genomes predicted that three strains did not encode any PAAR proteins in intact T6SS clusters. Another 12 strains with incomplete T6SS clusters were not included in the analysis; thus, a total of 226 strains were included in the analysis. The *paar* genes of most strains (158 of 226) were located in the variable region after *vgrG*. However, in other strains, the *paar* genes were behind *tssA* genes, and some (30 of 67) of them included a bacterial toxin 44 domain at the C-terminal. One strain, designated strain 47, had both kinds of PAAR-encoding genes in the intact T6SS cluster. Figure 8 shows the ML phylogenetic tree based on the amino acid sequences of the PAAR proteins. The PAAR proteins were separated into 10 groups, designated 1–10. The conservative domain of PAAR could be divided into two main types, namely, one type is PAAR with Ntox44 (pfam15607) and PAAR_CT_2 (cd14744) domains, while the other type only contains a PAAR_CT_2 domain. PAAR with bacterial toxin 44 at the C-terminus belongs to PAAR8 and PAAR9.

**FIGURE 9:**
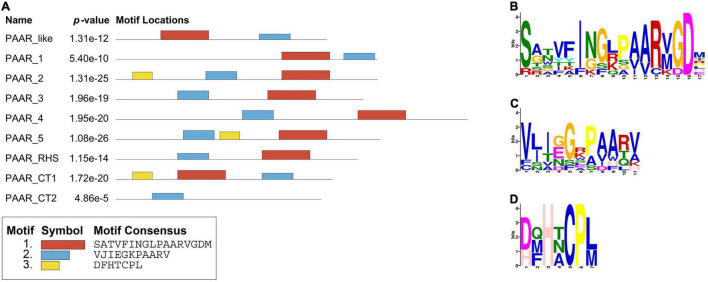
Protein motifs distribution of PAAR_like family members. **(A)** Motif compositions of PAAR_like family. The distributions of three conserved motifs are represented by different colored boxes. **(B–D)** Representative conserved amino acids of motifs 1–3. The amino acid position is shown on the *X*-axis, and the bit-score showing the probability of each of the amino acids at each position is shown on the *Y*-axis.

All the PAAR proteins in this analysis contained a PAAR-CT_2 domain and were members of the PAAR_like domain superfamily. This superfamily is subclassified into eight subgroups (cd14737–cd14744) based on the different predictive functions of the additional N- and C-terminal domains in the family members (Shneider et al., 2013). To further understand the characteristics of PAAR in *K. pneumoniae*, the MEME online program was utilized to analyze the motifs of different subgroups (as shown in Figure 9). Analysis of motif distributions showed that the length of the conserved motif varied from 7 to 17 amino acid residues, and all subgroups shared motif 2 (blue box). Compared with the other PAAR_like subgroups, the PAAR_CT_2 subgroup lacked motif 1 and motif 3, which might indicate that these subgroups have experienced divergence of gene functions.

### Alignment of Phospholipase D Proteins

**FIGURE 10:**
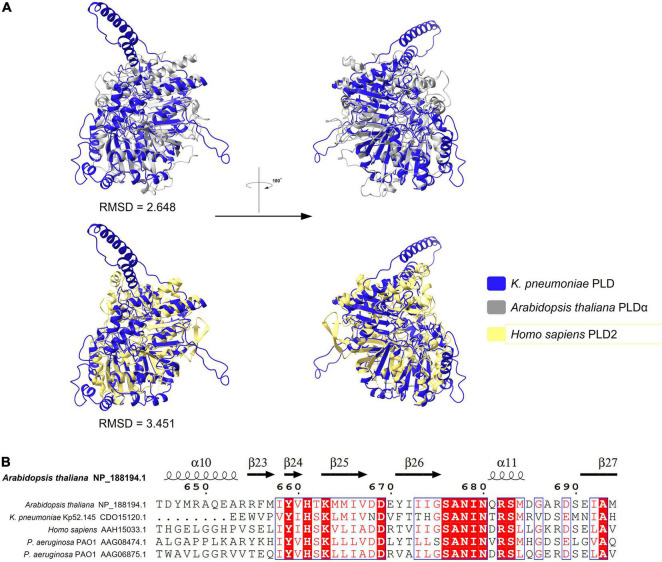
Predicted structure of *K. pneumoniae* PLD fold with analogous PLD domain. **(A)** Superimposition structure of *K. pneumoniae* PLD prediction structure (blue, predicted by AlphaFold2) with *Arabidopsis* PLDα (silvery, PDB:6KZ9), or human PLD2 catalytic domain structure (yellow, PDB:6OHM). **(B)** Sequence alignment among PLD proteins showing the HKD motif that are conserved. Refer to Supplementary Files 2 for raw multiple sequence alignment.

A novel virulence factor PLD encoded in a T6SS locus was previously reported in *K. pneumoniae* Kp52.145 and may be involved in the pathogenicity of this bacterial species. PLD in *K. pneumoniae* belongs to the cardiolipin synthase subfamily and plays a role in balancing phosphatidylglycerol (PG) and cardiolipin (Lery et al., 2014). However, whether it is secreted by T6SS has not been verified. BLASTp revealed that proteins of the PLD superfamily were identified in no more than 16 strains in this study. As stated above (Table 1), this is a class of Tle effector proteins with lipase function. PLD belongs to the PLD superfamily and is widely distributed in prokaryotes and eukaryotes. Members of the PLD superfamily usually contain a conserved region of the catalytic HKD motif, H-x-K-(x)4-D, where x is any amino acid (Metrick et al., 2020). The sequence alignment results of the PLD family protein in *K. pneumoniae* (by Phyre2 fold recognition server) demonstrated 26% identity with known PLD alpha (PLDα; PDB:6KZ9) of *Arabidopsis thaliana* and 25% identity with phospholipase D2 (PLD2) catalytic domain (PDB:6OHM) of *Homo sapiens*. The structure and quality assessment parameters for the *K. pneumoniae* PLD protein are shown in Supplementary File 3. Structural deviations were assessed by RMSD values. The RMSD between PLD in *K. pneumoniae* and PLDα or PLD2 was 2.648 and 3.451, respectively. The predicted structure of PLD in *K. pneumoniae* showed a highly similar overlap with PLDα and PLD2 (Figure 10A), indicating a conserved organization of these enzymes. The PLDA (AAG06875.1) and PLDB (AAG08474.1) effectors of the T6SS in *P. aeruginosa* PAO1 also belong to the PLD family of proteins. Therefore, a multiple-amino-acid-alignment outcome profile for the PLD proteins of different species was generated (Supplementary File 2). The alignment outcome reveals significant differences among the five protein sequences, and the catalytic HKD motif was highly conserved (Figure 10B). While these PLD proteins have not been well conserved in the primary sequences, their domain structures show similar topologies. In addition, the conserved nature of the HKD catalytic motifs implies that the members of the PLD superfamily have similar reaction mechanisms against the substrate.

## Discussion

The T6SS is a macromolecular protein export apparatus that is widely distributed in Gram-negative bacteria and is mainly involved in bacterial competition but also plays a role in pathogenicity and interactions with the environment (Monjaras Feria and Valvano, 2020; Wood et al., 2020; Yu et al., 2021). In the previously identified T6SSs, the number and sequence of genes encoded by different strains were variable. In this study, all strains of *K. pneumoniae* encoded a T6SS locus, and the number of loci ranged from 1 to 4, with most strains encoding two T6SS clusters. In addition, there were 12 strains in the study that did not contain any intact T6SS loci. Further studies are needed to determine whether the absence of some T6SS-related genes in some strains of *K. pneumoniae* affects the ability of the strains to exert normal T6SS functions. Moreover, the T6SS activity of each strain needs to be verified, regardless of whether there is an intact T6SS system gene cluster, because the T6SS strategy varies according to the environment. For example, *V. cholerae* attack indiscriminately with their T6SS (Stolle et al., 2021), while *P. aeruginosa* assembles and fires effectors only after detecting an initial attack from other bacteria, which is known as a T6SS tit-for-tat strategy (Basler et al., 2013; Smith et al., 2020). Furthermore, the T6SS activity of *A. baumannii* was reported to be inhibited in the presence of conjugative multidrug resistance plasmids (Di Venanzio et al., 2019).

Most isolates of *K. pneumoniae* in this study had a complete T6SS gene cluster that belonged to type i2 T6SS. Comparative analyses revealed that the type i2 T6SS in *K. pneumoniae* was conserved except for the variable region. In the T6SS loci of *K. pneumoniae*, proteins with a PAAR_CT_2 domain have been frequently found downstream of *vgrG* genes, while in some strains, genes encoding these PAAR proteins were found behind *tssA* genes. The variable region downstream of the *vgrG* gene varies widely between species and may encode putative effector-immune protein pairs. In *K. pneumoniae*, the *vgrG* gene is usually followed by multiple genes encoding immune proteins, then 1 or 2 genes encoding effectors, and then *paar* genes. The order and number of genes encoding effector-protein pairs vary from strain to strain. For example, in *Acinetobacter baumannii*, genes are identified in the order of effector and then immune protein (Lewis et al., 2019). Previous study has suggested that the variable region containing effector–immunity pairs differs from the origin of the T6SS gene cluster and may be acquired independently (Unterweger et al., 2014). In some strains, the assembly and secretion of T6SSs also require the participation of chaperone proteins (Manera et al., 2021). For example, DUF2169 protein (chaperone) is required for the antimicrobial activity of the Tde2 effector of *Agrobacterium tumefaciens* and for stabilization of the effector (Bondage et al., 2016). However, chaperones were not predicted in the vicinity of the effector-immune protein pairs in *K. pneumoniae*. Predicted chaperones have been found in other regions outside the T6SS gene cluster in some isolates of *K. pneumoniae*, but their functions are unknown. Understanding the role and interaction of chaperones in T6SSs is a knowledge gap that would markedly enhance the understanding of the dynamics of T6SSs.

To identify novel Tses in *K. pneumoniae*, protein sequences within and outside the T6SS gene cluster were analyzed using bioinformatics tools. In total, four types of effector proteins were identified, namely Tde, Tle, Tse, and Tpe, and the functions of these proteins include lipase, DNase/RNase, hydrogen peroxide catalysis, and polysaccharide hydrolase activities, respectively. Further work is needed to verify whether all the putative proteins are Tses. Among the identified effectors, groups 4, 5, 6, and 10 were located within the T6SS gene cluster. Proteins of group 4 had the same conserved DUF2235 domain as the toxin Hcp-ET identified in *E. coli* (Liu et al., 2017; Ma et al., 2017a). Overexpression of the effector gene *tle*^1KP^ in the periplasm inhibits the growth of *E. coli* (Liu et al., 2017). Group 5 is a class of effector proteins with an alpha/beta hydrolases domain, but the function of these proteins is unclear. The group 6 protein with a PLD domain may be a toxin targeting eukaryotic cells and may be involved in the pathogenesis of *K. pneumoniae* because the PLD mutant strain was avirulent in a mouse model of acute pneumonia (Lery et al., 2014). Group 10 is PAAR, which is one of the T6SS components. Previous reports have indicated that the PAAR-repeat family forms a sharp conical extension on the VgrG spike, a trimeric protein complex of the bacterial T6SS (Zheng et al., 2021). Most PAAR-repeat family contains C-terminal domain extensions corresponding to several uncharacterized domains, such as S-type pyocin, DUF2235, DUF2345, and cytotoxic proteins (Ma et al., 2017b; Liu et al., 2021). Some strains of *K. pneumoniae* in this study lacked a PAAR protein in the type i2 T6SS locus, and it is not yet known whether this will affect the T6SS function of these strains. In this study, all PAAR proteins of type i2 T6SS loci contained the PAAR_CT_2 domain, which is a member of the PAAR_like family. A small group of PAAR proteins in this study had a toxin 44 domain in the C-terminal extension region. The PLD superfamily is a large and diverse group of proteins, including plant, mammalian, and bacterial PLDs. Most members of this superfamily contain a short conserved sequence motif (H-x-K-x(4)-D, where x represents any amino acid residue), called the HKD signature motif (McDermott et al., 2020). In addition to the above four effector proteins, there are many identified proteins whose functions are unknown. The specific functions of these effector proteins require further investigation and characterization to improve the understanding of the functions of T6SSs and facilitate the resolution of clinical practical problems. For example, *Vibrio natriegens* was recently transformed into an effective antibacterial weapon by engineering a T6SS controlled by an externally induced on/off switch (Jana et al., 2021).

Because the T6SS is an effective bacterial weapon, mechanisms need to exist to avoid the bacteria poisoning itself. Therefore, T6SS-positive bacteria produce immunity proteins that interact directly with the effector proteins and inhibit their toxic activity. Immunity proteins that specifically antagonize toxins often appear in the vicinity of effectors. Effector-immune protein pairs were also found in *K. pneumoniae*; for example, an effector containing the DUF2235 domain was adjacent to the clusters of immune proteins (Figure 2). However, due to the limited data in the immune protein database, no immune proteins were identified around other predicted effector proteins. Bacteria can also withstand T6SS attacks through immunity protein-independent mechanisms (Flaugnatti et al., 2021). A previous study reported that T6SS orphan immunity gene clusters were present in the human gut microbiome and suggested that the acquisition and maintenance of orphan immune genes were a common mechanism for Bacteroidetes to resist bacterial antagonism in the human gut microbiome (Ross et al., 2019). Immune proteins, therefore, warrant further exploration in the future.

Considering the role of T6SSs in colony remodeling and pathogenicity and the current lack of knowledge about T6SSs in *K. pneumoniae*, bioinformatics methods were employed to analyze the T6SS and its effectors in 241 strains of *K. pneumoniae*. The T6SS genes in *K. pneumoniae* showed high diversity and potential plasticity at the species level. Future work will focus on the identification and characterization of these unknown functional effector proteins. Findings from this study provide a foundation for future studies of *K. pneumoniae* T6SSs and their effectors.

## Data Availability Statement

The datasets presented in this study can be found in online repositories. The names of the repository/repositories and accession number(s) can be found in the article/Supplementary Material.

## Ethics Statement

Samples used in this study were kindly provided by the Institute of Antibiotics, Huashan Hospital. The studies involving human participants were reviewed and approved by the Ethics Committee of Huashan Hospital, Fudan University, and Sir Run Run Shaw Hospital, Zhejiang University. Written informed consent to participate in this study was provided by the participants’ legal guardian/next of kin. The Informed Consent Form was waived by the Huashan Institutional Review Board if using strains for further study from the Culture Collection. Personal privacy is not involved in this study.

## Author Contributions

WL, XL, and JZ conceived and designed the work. WL conducted the bioinformatic analysis and wrote the manuscript. All authors read and approved the final manuscript.

## Conflict of Interest

The authors declare that the research was conducted in the absence of any commercial or financial relationships that could be construed as a potential conflict of interest.

## Publisher’s Note

All claims expressed in this article are solely those of the authors and do not necessarily represent those of their affiliated organizations, or those of the publisher, the editors and the reviewers. Any product that may be evaluated in this article, or claim that may be made by its manufacturer, is not guaranteed or endorsed by the publisher.

## Footnotes

1
http://www.ncbi.nlm.nih.gov/Structure/cdd/cdd.shtml

2
https://itol.embl.de/

3
https://github.com/tseemann/mlst

4
https://www.ncbi.nlm.nih.gov/cdd/

5
http://meme-suite.org/tools/meme

6
http://www.sbg.bio.ic.ac.uk/phyre2/html/page.cgi?id=index

7
https://www.genome.jp/toorls-bin/clustalw

8
http://espript.ibcp.fr/ESPript/ESPript/
