# Supplementary material for: Identification and Comparative Genomic Analysis of Type VI Secretion Systems and Effectors in Klebsiella pneumoniae
Source: Front Microbiol. 2022 May 12;13:853744. doi: 10.3389/fmicb.2022.853744 (PMC9134191; doi:10.3389/fmicb.2022.853744)
Supplement: Supplementary file 1 [file Data_Sheet_1.PDF]

Supplementary File S1a

(((SAMN13663554:0.000018075,((SAMN15052358:0.000066548,((((SAMN24664061:0.0000102
26,SAMN24669135:0.000008680)0.992:0.000011566,((((SAMN24665690:0.000014785,SAMN24
665694:0.000022334)0.853:0.000002797,((SAMN24667607:0.000006159,((((SAMN24667379:0.0
00008560,(SAMN24667610:0.000008607,(SAMN24665699:0.000006681,SAMN24665827:0.000
000874)0.934:0.000004532)0.893:0.000003019)0.756:0.000001500,SAMN24667608:0.000011625
)0.852:0.000003071,SAMN24667606:0.000002447)0.745:0.000001241,(SAMN24665696:0.00000
5130,SAMN24669138:0.000049603)0.998:0.000000005)0.384:0.000000005)0.505:0.000000005,S
AMN24669136:0.000017141)0.347:0.000000005)0.753:0.000001628,(SAMN24664235:0.000000
005,SAMN24669134:0.000011122)0.964:0.000004922)0.507:0.000003255,(SAMN24667378:0.00
0018544,SAMN24663454:0.000013570)1.000:0.000139168)0.959:0.000000005)1.000:0.00027093
9,(SAMN09977093:0.000010847,((((SAMN14132403:0.000007231,SAMN09988182:0.00000602
6)0.370:0.000000005,(SAMN10473953:0.000004820,(SAMN10433884:0.000000005,(SAMN122
28410:0.000006027,(SAMN13978114:0.000006026,((SAMN15186437:0.0,SAMN15186439:0.0,S
AMN15186441:0.0,SAMN15186442:0.0,SAMN15186445:0.0,SAMN15186446:0.0):0.000000005,
SAMN15186440:0.000003616)1.000:0.000078342)0.846:0.000002410)0.842:0.000000005)0.907:
0.000000006)0.938:0.000003615)0.624:0.000000005,SAMN16604318:0.000015667)0.760:0.0000
01205,SAMN18160617:0.000018081)0.760:0.000001205)0.282:0.000000005)1.000:0.000058380,
(((SAMN02152539:0.000756116,(((SAMN05412453:0.000006026,SAMN05412457:0.000000005
)1.000:0.000085008,(SAMN03024585:0.001806472,(SAMN06112197:0.000040876,SAMN07312
490:0.000071240)0.929:0.000006746)1.000:0.000140047)1.000:0.000083134,((SAMN03024610:0
.000168987,(((SAMN14168397:0.000003615,SAMN14168398:0.000002410)0.866:0.000003190,(
((SAMN14333445:0.0,SAMN14333447:0.0):0.000000005,SAMN14333446:0.000003615)1.000:0.
000020673,(SAMN11634268:0.000003799,(SAMN16418109:0.000007230,SAMN16418314:0.00
0000005)1.000:0.000552159)1.000:0.000200107)0.942:0.000006271)0.227:0.000007177,(SAMN1
6933298:0.000000005,SAMN16933304:0.000877671)1.000:0.000284206)1.000:0.000026807)0.66
0:0.000017778,SAMN08605586:0.000390554)1.000:0.000058856)0.595:0.000008236)0.728:0.00
0009278,(((SAMN10497376:0.002020893,(SAMN02602959:0.000107773,SAMN11047257:0.002
954232)1.000:0.000429999)1.000:0.000125144,((SAMN09692822:0.000099126,SAMN14421540:
0.000260415)1.000:0.000348203,(((((((((SAMN08391417:0.000026498,(SAMN07421000:0.0024
71117,((SAMN07421004:0.0,SAMN07421005:0.0):0.000000005,SAMN07421006:0.000808677)1
.000:0.000188741)1.000:0.000099431)1.000:0.001779351,(SAMN08135572:0.011031332,SAMN
13161584:0.001460281)1.000:0.000999254)1.000:0.000162971,(SAMN03024576:0.001605850,S
AMN09912217:0.001763051)1.000:0.000346052)1.000:0.000129578,((SAMN10986063:0.008212
989,(SAMN16933300:0.001774596,(SAMN10721859:0.001214765,SAMN17976248:0.00181742
5)1.000:0.000346063)0.342:0.000125219)1.000:0.000161832,(SAMEA3283117:0.002526389,((S
AMN06112206:0.000238216,SAMN16197931:0.000204205)1.000:0.000719651,(SAMN0963820
4:0.001094481,(SAMN07811429:0.000013256,(SAMN18106513:0.000196503,(SAMN15816123:
0.000026513,SAMN24665688:0.000225260)0.840:0.000000005)0.746:0.000001205)1.000:0.0007
66842)1.000:0.000600612)1.000:0.000408394)0.998:0.000177259)0.997:0.000094217)0.988:0.00
0080183,((SAMN10390469:0.001772496,(SAMN10740630:0.001226324,(SAMN03024573:0.000
627503,SAMN05928590:0.000732114)1.000:0.000518940)1.000:0.000444775)1.000:0.000172993
,(((SAMN24665686:0.001683789,((SAMN14402321:0.000520175,(SAMN12307651:0.000302813

,(SAMN17914336:0.000439141,(SAMN04008891:0.000182086,(SAMN04008892:0.001529440,S
AMN04008893:0.001404392)0.430:0.000000005)0.983:0.000009599)1.000:0.000678611)0.999:0.
000031626)1.000:0.000690905,(SAMN12877722:0.000486200,(SAMN02649400:0.000078738,S
AMN08380932:0.000735764)1.000:0.000256781)1.000:0.000211921)1.000:0.000786398)0.992:0.
000218107,(SAMN16085534:0.001549777,(SAMN06019523:0.000037364,SAMN12559024:0.00
0111100)1.000:0.001414842)0.997:0.000182689)1.000:0.000193894,((((SAMN04377969:0.00234
7226,SAMN08380941:0.002398870)1.000:0.000599743,(SAMN24664090:0.001631550,SAMN03
996301:0.001570826)1.000:0.000263691)0.367:0.000044255,(SAMN16246721:0.003097231,(SA
MN10956502:0.000533442,(SAMN06200334:0.000171184,(SAMD00060934:0.000630966,(((SA
MN11054834:0.000399715,(SAMN13503954:0.000002409,SAMN13503956:0.000227913)1.000:
0.000209806)0.966:0.000007214,SAMN08915824:0.000229090)0.313:0.000000005,(SAMN0523
1874:0.006318211,(SAMN16386297:0.000034965,(SAMN04377968:0.000024119,SAMN085823
15:0.000620414)0.753:0.000001205)1.000:0.000012052)0.084:0.000000005)0.999:0.000014463)0
.622:0.000000005)1.000:0.000077865)1.000:0.001275102)1.000:0.000191886)0.999:0.000113528,
(((((SAMN16933289:0.000007230,(SAMN16933269:0.000299019,SAMN16933305:0.000000005
)0.995:0.000008435)0.994:0.000009642,(SAMN05412452:0.000014461,(SAMN05412455:0.0000
02404,SAMN05412456:0.000109695)1.000:0.000210964)0.758:0.000001202)1.000:0.001229033,
((((SAMN18478563:0.000001206,((SAMN17915109:0.000003617,SAMN17915080:0.000067533
)0.935:0.000000005,((SAMN17911762:0.0,SAMN17915099:0.0):0.000003617,(SAMN17911845:
0.0,SAMN17911903:0.0,SAMN17912169:0.0,SAMN18478623:0.0):0.000000005)0.084:0.000000
005)0.000:0.000000005)0.562:0.000000005,SAMN18478480:0.000002411)1.000:0.000598034,(S
AMN07572687:0.000148157,SAMN08389138:0.000024499)1.000:0.000201284)1.000:0.0010005
94,(SAMN14402336:0.000909809,(SAMN04087461:0.000021698,SAMN04087462:0.000010849)
1.000:0.000181320)1.000:0.000744797)1.000:0.000460902)1.000:0.000244362,(SAMN16705163:
0.001501343,(SAMN07738870:0.001479772,((((SAMN14402341:0.000115724,SAMN14402356:
0.000009634)1.000:0.000305146,(SAMN04044480:0.000015661,(SAMN18096031:0.000042191,
SAMN10531975:0.014814641)0.818:0.000000005)0.386:0.000002379)1.000:0.000517472,(SAM
N24667609:0.000308798,SAMN04421276:0.000360628)0.878:0.000000005)1.000:0.000461050,(
SAMN05928542:0.000122389,((((SAMN11793565:0.007196137,(SAMN02885365:0.000020460,
SAMN08022168:0.000785971)1.000:0.000169959)0.800:0.000000005,SAMN14770898:0.000016
872)0.993:0.000011970,(SAMN14421541:0.000759608,((SAMN10956433:0.000149463,SAMN1
0963497:0.000045789)1.000:0.000226637,SAMN06112194:0.000137408)0.910:0.000015663)0.94
6:0.000004941)1.000:0.000056269,((SAMN24669140:0.000008500,(SAMN24668372:0.00000000
6,SAMN24668373:0.000002487)0.995:0.000012553)1.000:0.000278120,(SAMN10439690:0.0000
00005,SAMN10439712:0.000001205)0.987:0.000009888)1.000:0.000060321)0.511:0.000010817)
1.000:0.000874588)1.000:0.000771515)0.969:0.000199100)0.999:0.000115050)0.998:0.00012635
6,((SAMN12085403:0.000596656,(SAMN17915140:0.000001205,SAMN18478625:0.000000005)
1.000:0.000631458)1.000:0.001291532,((((SAMN24669141:0.000534712,SAMN16685806:0.001
030261)1.000:0.001035182,SAMN04244588:0.001921812)1.000:0.000231694,(SAMN08380938:
0.001770805,SAMN10724437:0.001756129)1.000:0.000357673)0.534:0.000059534,(((SAMN026
03941:0.003320937,(SAMN07602587:0.000244458,(((SAMN04917370:0.000570801,SAMN0802
6708:0.002252904)1.000:0.000347291,(((SAMN05412444:0.000004820,(SAMN05412796:0.0000
62671,((SAMN05412458:0.0,SAMN05412459:0.0,SAMN05412802:0.0,SAMN05412804:0.0):0.0
00000005,(SAMN05412803:0.000001205,SAMN05412797:0.000182023)0.935:0.000000005)0.88

2:0.000000005)0.935:0.000003615)1.000:0.000087981,(SAMN03770912:0.000008435,(SAMN15
946735:0.000001205,SAMN15946737:0.000004820)1.000:0.000016871)0.947:0.000004820)0.989
:0.000008436,(SAMN11835544:0.005800382,(SAMN05908511:0.000008435,SAMN14421539:0.
000119323)0.998:0.000010845)0.410:0.000000005)1.000:0.000000005)0.870:0.000001220,SAM
N04087463:0.000056653)1.000:0.000055809)1.000:0.001169915)1.000:0.000275362,(SAMN178
97699:0.001282780,SAMN08380935:0.001734606)0.969:0.000175930)0.000:0.000103970,(SAM
N16685805:0.000006027,((SAMN06112200:0.0,SAMN06112222:0.0):0.000156688,(SAMN06112
204:0.0,SAMN06112228:0.0):0.000002410)0.866:0.000002408)1.000:0.001602328)0.000:0.00008
0887)1.000:0.000110436)0.000:0.000050045)1.000:0.000411487)0.886:0.000086450)1.000:0.0001
00813)0.777:0.000069500)1.000:0.000130192,SAMN08380933:0.001253388)1.000:0.000242704,
(((SAMN06438459:0.000072235,SAMN15903654:0.000510500)1.000:0.000256909,((SAMN2466
5246:0.000539110,(SAMN11270035:0.000032538,SAMN16093148:0.000020486)1.000:0.000069
160)1.000:0.000607719,(SAMN05231873:0.001621775,SAMN14482422:0.010954536)1.000:0.00
0180334)0.996:0.000222970)1.000:0.000761867,SAMN05412805:0.002292951)1.000:0.00023018
2)1.000:0.000213767,(SAMN10740431:0.001655979,(SAMN02777842:0.000251133,SAMN0541
2811:0.000217196)1.000:0.000575522)1.000:0.000541452)1.000:0.000261503,SAMN03024590:0.
001589245)1.000:0.000573894)1.000:0.000073368)0.284:0.000012833,((SAMN10662038:0.0000
74878,(SAMN14402333:0.000591472,(SAMN06173548:0.001132278,SAMN06173549:0.000760
830)0.927:0.000005669)1.000:0.000025184)1.000:0.000390404,(SAMN03067424:0.000222261,((
SAMN02820977:0.000025305,(SAMN07503096:0.000009640,(SAMN07452764:0.000001205,SA
MN07503088:0.000040971)0.955:0.000004820)0.995:0.000012050)0.990:0.000014939,(SAMN06
437491:0.000014460,(SAMN11105635:0.000038560,(SAMN05149976:0.000003614,SAMN1693
3286:0.001031041)0.990:0.000009640)0.995:0.000010845)0.928:0.000006920)1.000:0.000098286
)1.000:0.000131849)1.000:0.000085767)1.000:0.000043834)0.976:0.000018556,((SAMN1124628
8:0.0,SAMN11246289:0.0):0.000004820,(SAMN11246290:0.0,SAMN11246291:0.0):0.000002410
)1.000:0.000156609)1.000:0.000086298)0.997:0.000020949)1.000:0.000054003,(SAMN09279554
:0.001157466,SAMN16246926:0.003173326)1.000:0.000414289)0.898:0.000000006)0.997:0.0000
00005,(SAMN12813532:0.000079570,SAMN07484315:0.000115816)1.000:0.000000005)0.952:0.
000000005,((SAMN07508215:0.000004821,(SAMN14411774:0.000243687,((SAMN14411773:0.
0,SAMN14411775:0.0):0.000000005,SAMN14411776:0.000000005)0.957:0.000000006)0.987:0.0
00007232)0.978:0.000007232,(SAMN06109054:0.000008436,((SAMN08380930:0.000095231,(S
AMN08380929:0.013690848,(SAMN08378909:0.000001205,SAMN08378686:0.000488592)0.74
3:0.000000005)0.734:0.000001198)1.000:0.000119339,(SAMN08378650:0.000001205,SAMN083
79292:0.000002410)0.950:0.000004820)0.702:0.000001205)0.771:0.000001205)0.793:0.00000120
5,((SAMN10434199:0.000081955,SAMN11602135:0.000012054)0.730:0.000001205,(SAMN076
72549:0.000008437,SAMN10107914:0.000364049)0.871:0.000002410)0.792:0.000000005);

Supplementary File S1b

(((SAMN24665246:0.000000005,(SAMN03024590:0.001373349,((SAMN24664061:0.0,SAMN24
664235:0.0,SAMN24665696:0.0,SAMN24665699:0.0,SAMN24665827:0.0,SAMN24667378:0.0,S
AMN24667379:0.0,SAMN24667606:0.0,SAMN24667607:0.0,SAMN24667608:0.0,SAMN246656
90:0.0,SAMN24665694:0.0,SAMN24667610:0.0,SAMN24669134:0.0,SAMN24663454:0.0,SAM
N24669135:0.0,SAMN24669136:0.0,SAMN24669138:0.0,SAMN02152539:0.0,SAMN02602959:
0.0,SAMN02777842:0.0,SAMN02820977:0.0,SAMN03024610:0.0,SAMN03067424:0.0,SAMN0
5149976:0.0,SAMN05412453:0.0,SAMN05412811:0.0,SAMN06109054:0.0,SAMN06112197:0.0,
SAMN07312490:0.0,SAMN07452764:0.0,SAMN07484315:0.0,SAMN07503088:0.0,SAMN0750
3096:0.0,SAMN07508215:0.0,SAMN07672549:0.0,SAMN08378650:0.0,SAMN08378686:0.0,SA
MN08378909:0.0,SAMN08379292:0.0,SAMN08380930:0.0,SAMN09279554:0.0,SAMN0969282
2:0.0,SAMN09977093:0.0,SAMN09988182:0.0,SAMN10107914:0.0,SAMN10433884:0.0,SAMN
10434199:0.0,SAMN10473953:0.0,SAMN10497376:0.0,SAMN10662038:0.0,SAMN11105635:0.
0,SAMN11246288:0.0,SAMN11246289:0.0,SAMN11246290:0.0,SAMN11246291:0.0,SAMN116
02135:0.0,SAMN11634268:0.0,SAMN12228410:0.0,SAMN12813532:0.0,SAMN13663554:0.0,S
AMN14402333:0.0,SAMN15052358:0.0,SAMN15186437:0.0,SAMN15186439:0.0,SAMN151864
40:0.0,SAMN15186441:0.0,SAMN15186442:0.0,SAMN15186445:0.0,SAMN15186446:0.0,SAM
N16246926:0.0,SAMN16604318:0.0,SAMN16933286:0.0,SAMN16933298:0.0,SAMN16933304:
0.0,SAMN18160617:0.0):0.000000005,(SAMN16418109:0.0,SAMN16418314:0.0):0.001369495)
0.782:0.001371050)0.927:0.004114951)0.838:0.001370169,(SAMN12085403:0.0,SAMN1791514
0:0.0,SAMN18478625:0.0):0.001372241)0.000:0.000000005,(SAMN04377969:0.001372432,SA
MN04244588:0.001371325)0.000:0.000000005,(((SAMN05412805:0.000000005,SAMN1608553
4:0.004135641)0.856:0.001371267,(((SAMN04087463:0.000000005,((SAMN03770912:0.0,SAM
N04917370:0.0,SAMN05412444:0.0,SAMN05412796:0.0,SAMN07602587:0.0,SAMN08026708:
0.0,SAMN11835544:0.0,SAMN15946735:0.0,SAMN15946737:0.0):0.000000005,SAMN0590851
1:0.000000005)0.861:0.001377226)0.790:0.001378305,((SAMN07421000:0.0,SAMN07421004:0.
0,SAMN07421005:0.0,SAMN07421006:0.0,SAMN08391417:0.0):0.005500974,SAMN08380935:
0.001373978)0.756:0.001374553)0.961:0.004130848,((SAMN06112200:0.0,SAMN06112204:0.0,
SAMN06112222:0.0,SAMN06112228:0.0,SAMN16685805:0.0):0.004122056,((((SAMN1072185
9:0.0,SAMN16933300:0.0):0.005638994,(((SAMN06019523:0.0,SAMN12559024:0.0):0.0013818
23,SAMN10986063:0.001379290)0.939:0.004139857,(SAMN12307651:0.001375727,(SAMN246
69141:0.0,SAMN02649400:0.0,SAMN04008892:0.0,SAMN04008893:0.0,SAMN08380932:0.0,S
AMN12877722:0.0,SAMN14402321:0.0,SAMN16685806:0.0,SAMN17914336:0.0):0.000000005
)0.964:0.005534981)0.838:0.002663332)0.998:0.048205501,((((SAMD00060934:0.0,SAMN04377
968:0.0,SAMN05231874:0.0,SAMN06200334:0.0,SAMN08582315:0.0,SAMN08915824:0.0,SA
MN10956502:0.0,SAMN11054834:0.0,SAMN13503954:0.0,SAMN13503956:0.0,SAMN1638629
7:0.0):0.014804939,((SAMN10740630:0.001333005,((SAMN03024573:0.000000005,SAMN0592
8590:0.000000005)0.981:0.006885815,(SAMN15903654:0.000000005,(SAMN06438459:0.00137
0606,(SAMN11270035:0.005479722,SAMN16093148:0.000000005)0.768:0.001371015)0.977:0.0
06947650)0.793:0.001375202)0.764:0.001406822)0.971:0.013150812,(((SAMN10390469:0.00547
6227,SAMN02603941:0.001367898)0.000:0.000000005,(SAMN17976248:0.004081470,(SAMN0
3996301:0.006810650,((SAMN08380933:0.000000005,(SAMN05231873:0.000000005,SAMN144
82422:0.001365726)0.990:0.008220328)0.867:0.002805886,(SAMN24664090:0.005472110,SAM

N03024576:0.001351227)0.899:0.004029661)0.196:0.001372926)0.753:0.001422114)0.949:0.004
127051)0.878:0.002728035,(SAMN07572687:0.000000005,((((((SAMN12085403:0.0,SAMN1791
5140:0.0,SAMN18478625:0.0):0.002675846,SAMN16933300:0.001436877)0.855:0.002804667,((
SAMN08380935:0.005265246,SAMN10986063:0.001591393)0.891:0.004140144,SAMN0773887
0:0.003900960)0.803:0.002901187)0.803:0.002383585,((((SAMN06112222:0.0,SAMN06112228:
0.0,SAMN16685805:0.0,SAMN06112200:0.0,SAMN06112204:0.0):0.004113871,SAMEA328311
7:0.006866347)0.905:0.004030195,SAMN17976248:0.001486526)0.739:0.001671654,SAMN107
21859:0.003792355)0.988:0.009685176)0.000:0.000000005,SAMN16705163:0.003172001)1.000:
0.759968565,(SAMN08380941:0.001718301,((SAMN24669140:0.0,SAMN02885365:0.0,SAMN0
5928542:0.0,SAMN08022168:0.0,SAMN10439690:0.0,SAMN10439712:0.0,SAMN10956433:0.0,
SAMN10963497:0.0,SAMN11793565:0.0,SAMN14421541:0.0,SAMN14770898:0.0,SAMN2466
8373:0.0,SAMN24668372:0.0,SAMN04044480:0.0,SAMN04421276:0.0,SAMN06112194:0.0,SA
MN06437491:0.0,SAMN14402341:0.0,SAMN14402356:0.0,SAMN18096031:0.0,SAMN2466760
9:0.0):0.004181539,SAMN03024590:0.000000005)0.774:0.002463472)1.000:0.346680265)1.000:
0.270438849)0.347:0.002749700)0.868:0.006205053)0.881:0.009458440)0.986:0.038417200,SA
MN10963497:0.100433385)0.998:0.097079255,(SAMN16705163:0.000000006,(SAMN16093148:
0.011459383,SAMN08380929:0.068700625)0.698:0.002595094)0.897:0.009152691)0.883:0.0339
07285)0.851:0.008820694,((SAMN09638204:0.001371811,(SAMN06112206:0.0,SAMN1619793
1:0.0):0.000000005)0.771:0.001370989,(SAMN10740431:0.004112136,SAMN08380938:0.01244
2075)0.740:0.001392067)0.754:0.002757080)0.784:0.002837719)0.000:0.000000005)0.959:0.004
121382)0.000:0.000000005,SAMN13161584:0.000000005)0.000:0.000000005);

Supplementary File S1c

((SAMN16933300:0.016073466,((SAMN12307651:0.0,SAMN04008891:0.0,SAMN04008893:0.0,
SAMN04008892:0.0,SAMN17914336:0.0,SAMN14402321:0.0):0.000000006,((SAMN16685805:
0.0,SAMN04377969:0.0,SAMN14168398:0.0,SAMN15186442:0.0,SAMN06112204:0.0,SAMN0
5412453:0.0,SAMN10473953:0.0,SAMN05412805:0.0,SAMN15946735:0.0,SAMN11602135:0.0,
SAMN15186437:0.0,SAMN14333445:0.0,SAMN08378650:0.0,SAMN14421540:0.0,SAMN1518
6441:0.0,SAMN03024590:0.0,SAMN14132403:0.0,SAMN08380929:0.0,SAMN09638204:0.0,SA
MN18478625:0.0,SAMN08380935:0.0,SAMN05412802:0.0,SAMN05412797:0.0,SAMN0742100
6:0.0,SAMN07421005:0.0,SAMN16418314:0.0,SAMN11246290:0.0,SAMN15186440:0.0,SAMN
08379292:0.0,SAMN13978114:0.0,SAMN14333447:0.0,SAMN14402333:0.0,SAMN15052358:0.
0,SAMN24665694:0.0,SAMN24664061:0.0,SAMN08380930:0.0,SAMN12085403:0.0,SAMN021
52539:0.0,SAMN05412811:0.0,SAMN06112197:0.0,SAMN05412796:0.0,SAMN09988182:0.0,S
AMN05412458:0.0,SAMN08380938:0.0,SAMN06173549:0.0,SAMN05412459:0.0,SAMN104973
76:0.0,SAMN07508215:0.0,SAMN24667378:0.0,SAMN16933298:0.0,SAMN14411775:0.0,SAM
N16197931:0.0,SAMN08378909:0.0,SAMN08026708:0.0,SAMN03067424:0.0,SAMN16418109:
0.0,SAMN13663554:0.0,SAMN10740431:0.0,SAMN05412444:0.0,SAMN03770912:0.0,SAMN0
3024585:0.0,SAMN15946737:0.0,SAMN24669134:0.0,SAMN24665827:0.0,SAMN07312490:0.0,
SAMN05412457:0.0,SAMN06173548:0.0,SAMN15186439:0.0,SAMN14411776:0.0,SAMN1433
3446:0.0,SAMN10107914:0.0,SAMN15186445:0.0,SAMN17915140:0.0,SAMN05908511:0.0,SA
MN06112228:0.0,SAMN07503088:0.0,SAMN09279554:0.0,SAMN16085534:0.0,SAMN1281353

2:0.0,SAMN06112206:0.0,SAMN07421004:0.0,SAMN02602959:0.0,SAMN09977093:0.0,SAMN
11835544:0.0,SAMN24664235:0.0,SAMN14411773:0.0,SAMN05149976:0.0,SAMN12228410:0.
0,SAMN24669135:0.0,SAMN16933304:0.0,SAMN15186446:0.0,SAMN08391417:0.0,SAMN246
67610:0.0,SAMN07672549:0.0,SAMN07421000:0.0,SAMN05412804:0.0,SAMN24665696:0.0,S
AMN24669136:0.0,SAMN13161584:0.0,SAMN03024610:0.0,SAMN10662038:0.0,SAMN246634
54:0.0,SAMN04917370:0.0,SAMN11246289:0.0,SAMN14411774:0.0,SAMN16933286:0.0,SAM
N11047257:0.0,SAMN07452764:0.0,SAMN24667379:0.0,SAMN11634268:0.0,SAMN06112222:
0.0,SAMN24667606:0.0,SAMN08605586:0.0,SAMN07602587:0.0,SAMN04244588:0.0,SAMN0
6112200:0.0,SAMN24665690:0.0,SAMN16246926:0.0,SAMN11246291:0.0,SAMN10434199:0.0,
SAMN24665699:0.0,SAMN14421539:0.0,SAMN02777842:0.0,SAMN07484315:0.0,SAMN1124
6288:0.0,SAMN09692822:0.0,SAMN10433884:0.0,SAMN16604318:0.0,SAMN08378686:0.0,SA
MN11105635:0.0,SAMN18160617:0.0,SAMN24669138:0.0,SAMN24667607:0.0,SAMN0541280
3:0.0,SAMN24667608:0.0,SAMN02820977:0.0,SAMN06109054:0.0,SAMN04087463:0.0,SAMN
06437491:0.0,SAMN07503096:0.0,SAMN14168397:0.0):0.143482611,(SAMN16933289:0.44442
4759,((SAMN10724437:0.0,SAMN02603941:0.0,SAMN16386297:0.0,SAMN13503956:0.0,SAM
N13503954:0.0,SAMN08915824:0.0,SAMN03996301:0.0,SAMN04377968:0.0,SAMN06200334:
0.0,SAMN10956502:0.0,SAMD00060934:0.0,SAMN11054834:0.0,SAMN08582315:0.0,SAMN0
5231874:0.0,SAMN24665246:0.0,SAMN15903654:0.0,SAMN16093148:0.0,SAMN05231873:0.0,
SAMN14482422:0.0,SAMN11270035:0.0,SAMN06438459:0.0,SAMN10740630:0.0,SAMN0302
4576:0.0,SAMN08380933:0.0,SAMN05928590:0.0,SAMN03024573:0.0,SAMN17976248:0.0,SA
MN10390469:0.0,SAMN24664090:0.0,SAMN17897699:0.0,SAMN04421276:0.0,SAMN0404448
0:0.0,SAMN14402356:0.0,SAMN14402341:0.0,SAMN10531975:0.0,SAMN24667609:0.0):0.000
000005,(SAMN14402336:0.000000005,(SAMN14770898:0.0,SAMN10439690:0.0,SAMN184785
63:0.0,SAMN11793565:0.0,SAMN02885365:0.0,SAMN05928542:0.0,SAMN17911903:0.0,SAM
N06112194:0.0,SAMN18478480:0.0,SAMN17915109:0.0,SAMN24669140:0.0,SAMN10956433:
0.0,SAMN18478623:0.0,SAMN10439712:0.0,SAMN08389138:0.0,SAMN24668372:0.0,SAMN2
4668373:0.0,SAMN17915080:0.0,SAMN08022168:0.0,SAMN17912169:0.0,SAMN17915099:0.0,
SAMN14421541:0.0,SAMN17911762:0.0,SAMN17911845:0.0,SAMN05412455:0.0,SAMN05412
456:0.0,SAMN05412452:0.0,SAMN16933305:0.0,SAMN16933269:0.0,SAMN07572687:0.0,SA
MN10963497:0.0):0.015377985)0.058:0.000002718)1.000:1.110649543)0.835:0.345249736)0.901
:0.188491832)0.872:0.016130928)0.000:0.000000005,(SAMN02649400:0.0,SAMN24669141:0.0,
SAMN08380932:0.0,SAMN12877722:0.0,SAMN16685806:0.0,SAMN16705163:0.0):0.00000000
5,(SAMN10721859:0.016132590,(SAMN06019523:0.0,SAMN12559024:0.0):0.015895008)0.434:
0.000000005);
