# Supplementary material for: Identification and Comparative Genomic Analysis of Type VI Secretion Systems and Effectors in Klebsiella pneumoniae
Source: Front Microbiol. 2022 May 12;13:853744. doi: 10.3389/fmicb.2022.853744 (PMC9134191; doi:10.3389/fmicb.2022.853744)
Supplement: Supplementary file 2 [file Data_Sheet_2.PDF]

```
Arabidopsis_thaliana_NP_188194       β1
                                     1         10        20        30        40        50
1 Arabidopsis_thaliana_NP_188194     .........MAQHLLHGTLHATIYEVDALHGGGVRQGFLGKILANVEETIGVGKGETQLY
2 K._pneumoniae_Kp52.145_CDO1512     ................MTQEDIITPIATVDTRQCMITSPWFVQNTEYSPMP.....ATYK
3 Homo_sapiens_AAH15033.1            MTATPESLFPTGDELDSSQLQMESDEVDTLKEGEDPADRMHPFLAIYELQSLKVHPLVFA
4 P._aeruginosa_PAO1_AAG08474.1      ..............MSDLYKPQTLKLYAQQAGSVRLTLDWFANKAFYPPRAG....VHIK
5 P._aeruginosa_PAO1_AAG06875.1      .....MLQKKPYNGLHEKELNQINQQDGSPCVAISAPGCFIKGSNLFSEKRAGNRVRFFT

Arabidopsis_thaliana_NP_188194       β2 TT β3           β4          β5       TT   .....
                                            60        70        80        90        100
1 Arabidopsis_thaliana_NP_188194     ATIDLQKARVGRTRKIKNEPKNPKWYESFHIYCAHLASDIIFTVKDDNPIGATLI.....
2 K._pneumoniae_Kp52.145_CDO1512     PLVNGEEAFAAVYHAIMNAQ......KTVDIICWGFQPSMYFIRDG..............
3 Homo_sapiens_AAH15033.1            PGVPVTAQVVGTERYTSGSKVGTCTLYSVRLTHGDFSWTTKKKYRHFQELHRDLLRHKVL
4 P._aeruginosa_PAO1_AAG08474.1      PLINGQAAFDAVHAAMEAAR......HSIDIITWGFDPAMRFKRPD..............
5 P._aeruginosa_PAO1_AAG06875.1      TGRDYFSDLASALDSASSSIFITGWQVNYDVLLDGRRSLWQCLRQALERSPALKVYVMPW

Arabidopsis_thaliana_NP_188194       ......... β6  η1   β7   TT   TT   β8   η2
                                             110       120       130       140       150
1 Arabidopsis_thaliana_NP_188194     .........GRAYIPVDQVINGEEVDQWVEILDNDRNPIQGGSKIHVKLQYFHVEEDRNW
2 K._pneumoniae_Kp52.145_CDO1512     .........QSLCIGELLCKIAETKKVQVRILGWEMPCNAAGVGGEANLPGKGVIRYKDR
3 Homo_sapiens_AAH15033.1            MSLLPLARFAVAYSPARDAGNREMPSLPRAGPEGSTRHAASKQKYLENYLNCLLTMSFYR
4 P._aeruginosa_PAO1_AAG08474.1      .........GPRIGELLQTKGREGVQARVLVWSNQLARLKENTIPGAGVGGSGGTWVGSG
5 P._aeruginosa_PAO1_AAG06875.1      LS.......PSGSLGTYDFETMLAVFQLNAGLEGGARAFCTPAIQQSDMQGLGVAFSHHQ

Arabidopsis_thaliana_NP_188194       TT   β9   TT   TT
                                        160       170       180       190       200       210
1 Arabidopsis_thaliana_NP_188194     N...MGIKSAKFPGVPYTFFSQRQGCKVSLYQDAHIPDNFVPRIPLAGGKNYEPQRCWED
2 K._pneumoniae_Kp52.145_CDO1512     K...GQSTTDERYAYDRQWFRQYS...LSGEWSDHQLKK.....................
3 Homo_sapiens_AAH15033.1            N...YHAMTEFLEVSQLSFIPDLGRKGLEGMIRKRSGGHRVPGLTCCGRDQVCYRWSKRW
4 P._aeruginosa_PAO1_AAG08474.1      V...ASGSAVDNEVLRLEQRRQHN...LNLIARQQEALERSERLHREGRLPSFDPRGAAH
5 P._aeruginosa_PAO1_AAG06875.1      KSVVIDNRIGYVGGIDLAYGRRDDNDFSLDASGRRGNDAYNPGLPHLGWMAEDEHVSSMG

Arabidopsis_thaliana_NP_188194       α1   β10 ............   TT   TT   α2
                                          220       230       240       250       260
1 Arabidopsis_thaliana_NP_188194     IFDAISNAKHLIYIT............GWSVYAEIALVRDSRRPKPGGDVTIGELLKKKA
2 K._pneumoniae_Kp52.145_CDO1512     GQAGIAEIAAAPIAQ............RQEKLSSLSPLFVG...................
3 Homo_sapiens_AAH15033.1            LVVKDSFLLYMCLETG...........AISFVQLFDPGFEVQVGKRSTEARHGVRIDTSH
4 P._aeruginosa_PAO1_AAG08474.1      ARTRIAELEAENAEI............QRTLDSSGAQGYGGKRGSGGTRQDPWGQIFTRD
5 P._aeruginosa_PAO1_AAG06875.1      LMMATLFDLSRPLASLTLHAPTLRLSPFPHIAASDEPLLSIPLAPSRARALNGAAYLSDL

Arabidopsis_thaliana_NP_188194       β11   η3   α3   α4   β12
                                         270       280       290       300       310       320
1 Arabidopsis_thaliana_NP_188194     SEGVRVLLLVWDDRTSVDVLKKDGLMATHDEETENFFRGSDVHCILCPRNPDDGGSIVQS
2 K._pneumoniae_Kp52.145_CDO1512     ...............RGFNFLERAEIAYRAANMALDP................DISPDTM
3 Homo_sapiens_AAH15033.1            RSLILKCSSYRQARWWAQEITELAQGPGRDFLQLHRHDSYAPPRPGTLARWFVNGAGYFA
4 P._aeruginosa_PAO1_AAG08474.1      WFKAVRGGGLQNVEFRTRDFEQTARPVMNGEQVRLVNGRLQSLIHLLRADGIDDLGIGQL
5 P._aeruginosa_PAO1_AAG06875.1      FRSPMLPSLQWLGRAYNSSKEGLDEGFERLDALRRQMVASSIRAIANLIADNLDALPIEP

Arabidopsis_thaliana_NP_188194       β13   β14   TT   TT
                                         330       340       350       360       370       380
1 Arabidopsis_thaliana_NP_188194     LQISTMFTHHQKIVVVDSEMPSRGGSEMRRIVSFVGGIDLCDGRYDTPFHSLFRTLDT..
2 K._pneumoniae_Kp52.145_CDO1512     LTLAGTVTHHQKTVLVDYELPES.......AVGFVMGHNMLDEYWDTDKHSAL.......
3 Homo_sapiens_AAH15033.1            AVADAILRAQEEIFITDWWLSPEVYLKRPAHSDDWRLDIMLKRKAEEGVRVSILLFKE..
4 P._aeruginosa_PAO1_AAG08474.1      LVLTQFASHHQKMVLVDYGSPQ........AIGFVMGHNMHRNYWDTSAHLFD.......
5 P._aeruginosa_PAO1_AAG06875.1      ELERRLRAWLEELRTAALNLPEALRIKSLLLINQWMSETELGQVLTLISGKGFEDIPQNL

Arabidopsis_thaliana_NP_188194       TT TT   η4   β15
                                                                   390       400
1 Arabidopsis_thaliana_NP_188194     ................................VHHDDFHQPNFTGAAITKGGPREPWHDI
2 K._pneumoniae_Kp52.145_CDO1512     ......................................FRPGNNMDPRLGANGKLPRQDI
3 Homo_sapiens_AAH15033.1            ................................VELALGINSGYSKRALMLLHPNIKVMRH
4 P._aeruginosa_PAO1_AAG08474.1      ......................................DRAAGRDP......GFGPWQDI
5 P._aeruginosa_PAO1_AAG06875.1      SGKAGELAGSLFWTLHRLMQARAGGHQQPYRYLDEAPQPLASPDNARLAADQPRMPWQDV

Arabidopsis_thaliana_NP_188194       β16   α5   η5   TT TT   β17
                                     410       420       430       440       450       460
1 Arabidopsis_thaliana_NP_188194     HSRLEGPIAWDVMYNFEQRWSKQGGKDILVKLRDLSDIIITPSPVMFQEDHDVWNVQLFR
2 K._pneumoniae_Kp52.145_CDO1512     SSRVTGPILEHLHHNFAMAWEKETGQDLLTIRDSVSIAK....KLKLRALHGTPVMAQLL
3 Homo_sapiens_AAH15033.1            PDQVTLWAHHEKLLVVDQVVAFLGGLDLAYGRWDDLHYRLTDLGDSSESAASQPPTPRPD
4 P._aeruginosa_PAO1_AAG08474.1      SMQVQGPVLADLSRNFSEAWDLETPWYKRWFSTPSLTAERDALPLPKIATPASNSVAQIC
5 P._aeruginosa_PAO1_AAG06875.1      HCRIEGPSVYDLARNFIDRWNGQQAYLAKTPALQDTALVRSALEAVMKWLNSLAAAAGLE
```

```
Arabidopsis_thaliana_NP_188194   α6  β18  β19  α7  β20
                                 470        480        490        500        510

1 Arabidopsis_thaliana_NP_188194  .SIDGGAAAGFPESPEAAAAEGLVSGKDNIIDRSIQDAYIHAIRRAKDFIYV........
2 K._pneumoniae_Kp52.145_CDO1512  .RTQAQAGKHDIETLYLQAVNNATQFIYIENQYFRWPPLAELINQVAERQSK........
3 Homo_sapiens_AAH15033.1         .SPATPDLSHNQFFWLGKDYSNLITKDWVQLDRPFEDFIDRETTPRMPWRDVGVVVHGLP
4 P._aeruginosa_PAO1_AAG08474.1   .RTQPQDDERSILEHYLKALGNATDYVYMENQYFRYAGFAERLRKTAQVRKA........
5 P._aeruginosa_PAO1_AAG06875.1   NYLDEKRNLRLELDPPTPCWINAPEQLPQEPEVRRGGMTVQVLRSAAARMLEQEQAGRLG

Arabidopsis_thaliana_NP_188194   η6  η7  α8  β21
                                 520        530        540        550        560        570

1 Arabidopsis_thaliana_NP_188194  ...ENQYFLGSSFAWAADGITPEDINALHLIPKELSLKIVSKIEKG....EKFRVYVVVP
2 K._pneumoniae_Kp52.145_CDO1512  ...VGRELHLFVVTNVTDEGIGAGTVNTQRMLEVLGRANIIPEVTK....LRKIGQLSN.
3 Homo_sapiens_AAH15033.1         ARDLARHFIQRWNFTKTTKAKYKTPIYPYLLPKSTSTANQLPFTLPGGQCTTVQVLRSVD
4 P._aeruginosa_PAO1_AAG08474.1   ...RGVPGDLYLFVVTNTPDSSDASKTTYDMMKGLGQEQLMPQVQR....DLAHDLREKR
5 P._aeruginosa_PAO1_AAG06875.1   AGVNLPLQVGVSTEGVQSNCKDAMLLAISGAQQFIYIENQFFQSEFGKEGEVFKDLPLSG

Arabidopsis_thaliana_NP_188194   α9
                                 580        590        600

1 Arabidopsis_thaliana_NP_188194  MWPEGLPESGSVQAILDWQRR.....................TMEMMYKDVIQALRAQ
2 K._pneumoniae_Kp52.145_CDO1512  ATFGGSVGYIDPGDINKRNRE.....................MSEKIADFKKKADEIQ
3 Homo_sapiens_AAH15033.1         RWSAGTLENSILNAYLHTIRESQHFLYIENQFFISCSDGRTVLNKVGDEIVDRILKAHKQ
4 P._aeruginosa_PAO1_AAG08474.1   EQLKQVRENLHPDPYVRRGQE.....................NNIERLERKIEALEEK
5 P._aeruginosa_PAO1_AAG06875.1   PMASLRDVGSLRRDFVVRIRLEEALEQRDLWLLDWAEVEKIAQEPGTEARQFLKSMLAMW

Arabidopsis_thaliana_NP_188194   η8  β22  TT
                                 610        620        630        640

1 Arabidopsis_thaliana_NP_188194  GLEEDPRN.....................YLTFFCLGNREVKKDGEYEPAEKP......
2 K._pneumoniae_Kp52.145_CDO1512  SSEILPEE.............................RPGLKVHICSLVAPDSPP......
3 Homo_sapiens_AAH15033.1         GWCYRVYVLLPLLPGFEGDISTGGGNSIQAILHFTYRTLCRGEYSILHRLKAAMG.....
4 P._aeruginosa_PAO1_AAG08474.1   GVTPEVEQRLGDLGAQEIPGLAKNTGEDDKPYQLVEVPGLKVVVATLATSDPAPG.....
5 P._aeruginosa_PAO1_AAG06875.1   GVNAQGWLTHKLGEAQHGLLNEIGEALARRIERAIQREHPFHVYLVLPVHPEGALNVPNI

Arabidopsis_thaliana_NP_188194

1 Arabidopsis_thaliana_NP_188194  ............................................................
2 K._pneumoniae_Kp52.145_CDO1512  ............................................................
3 Homo_sapiens_AAH15033.1         .........................................................TAW
4 P._aeruginosa_PAO1_AAG08474.1   .........................................................SPP
5 P._aeruginosa_PAO1_AAG06875.1   MHQVHLTQQSLVFGEQSLVKRIQRQMALKALEGKSDPAQAREIIERKDARGRPVYEQQDW

Arabidopsis_thaliana_NP_188194   α10  β23  β24  β25  β26  α11  β27
                                 650        660        670        680        690

1 Arabidopsis_thaliana_NP_188194  .......DPDTDYMRAQEARRFMIYVHTKMMIVDDEYIIIGSANINQRSMDGARDSEIAM
2 K._pneumoniae_Kp52.145_CDO1512  ..................EEWVPVYIHSKLMIVNDVFTTHGSANINTRSMRVDSEMNIAH
3 Homo_sapiens_AAH15033.1         RDYISICGLRTHGELGGHPVSELIYIHSKVLIADDRTVIIGSANINDRSLLGKRDSELAV
4 P._aeruginosa_PAO1_AAG08474.1   PARLSAEAEAALGAPPLKARYKHIYVHSKLLLVDDLYTLLSSANINVRSMHGDSELGVAQ
5 P._aeruginosa_PAO1_AAG06875.1   SRYLTLLNLRTWAVLGGRVVTEQIYVHSKLLIADDRVAILGSANINDRSLQGERDSELAV

Arabidopsis_thaliana_NP_188194   TT  α12  η9  α13
                                 700        710        720        730        740

1 Arabidopsis_thaliana_NP_188194  GGYQPHHLSHRQPARGQIHG......FRMSLWYEHLGMLDETFLDPSSLECIEKVN....
2 K._pneumoniae_Kp52.145_CDO1512  ...........EWSSVTRD......LRRRLWNMHTNGRGGQDD...PAKAFEEWG....
3 Homo_sapiens_AAH15033.1         LIEDTETEPSLMNGAEYQAG......RFALSLRKHCFGVILGANTRPDLDLRDPIC....
4 P._aeruginosa_PAO1_AAG08474.1   ...........PNPDLARA......MREELWELHAQKVATT.....TEKNFKLWN....
5 P._aeruginosa_PAO1_AAG06875.1   MVRDSEPLTVRLDGKNDAIVGKAIHQLRVNLWKKHFGLSQGPGGFVKPASELSAYLSIPA

Arabidopsis_thaliana_NP_188194   β28  β29  TT  β30  TT  TT  TT
                                 750        760        770        780        790        800

1 Arabidopsis_thaliana_NP_188194  .RISDKYWDFYSSESLEHDLPGHLLRYPIGVASEGDITELPGFEFFPDTKARILGTKSDY
2 K._pneumoniae_Kp52.145_CDO1512  .YILKENKDLQGTKKNKPVASLIKFFYNKSTLSDLD........................
3 Homo_sapiens_AAH15033.1         .DDFFQLWQDMAESNANIYEQIFRCLPSNATRSLRTLREYVAVEPLATVSPPLARSELTQ
4 P._aeruginosa_PAO1_AAG08474.1   .QKMDANWRQQ.RKDEPLVANLLRFWDVVTPYSPGLTVD.....................
5 P._aeruginosa_PAO1_AAG06875.1   AQEAWEAIQTLAKENTRAYERTFNFIPQNISQTQLQLTPEPPKGFEDGFPASIWPTWAYR

Arabidopsis_thaliana_NP_188194   α14
                                 810

1 Arabidopsis_thaliana_NP_188194  LPPILTT.....................................................
2 K._pneumoniae_Kp52.145_CDO1512  ............................................................
3 Homo_sapiens_AAH15033.1         VQGHLVHF...............PLKFLEDESLLPPLGSKEGMIPLEVWT..........
4 P._aeruginosa_PAO1_AAG08474.1   ............................................................
5 P._aeruginosa_PAO1_AAG06875.1   KPGELRAGGQLMEPMPYQEIFWRSSNLTSVKTFPPPNGVSGFITALPTSWTRGERNDSGL
```

Arabidopsis_thaliana_NP_188194

```
1 Arabidopsis_thaliana_NP_188194  ...............................
2 K._pneumoniae_Kp52.145_CDO1512  ...............................
3 Homo_sapiens_AAH15033.1         ...............................
4 P._aeruginosa_PAO1_AAG08474.1   ...............................
5 P._aeruginosa_PAO1_AAG06875.1   NLSILAHQDSRSLPTQVAMNGDSSAQGKHRT
```
