# Supplementary figures and images for: Identification and Comparative Genomic Analysis of Type VI Secretion Systems and Effectors in Klebsiella pneumoniae
Source: Front Microbiol. 2022 May 12;13:853744. doi: 10.3389/fmicb.2022.853744 (PMC9134191; doi:10.3389/fmicb.2022.853744)

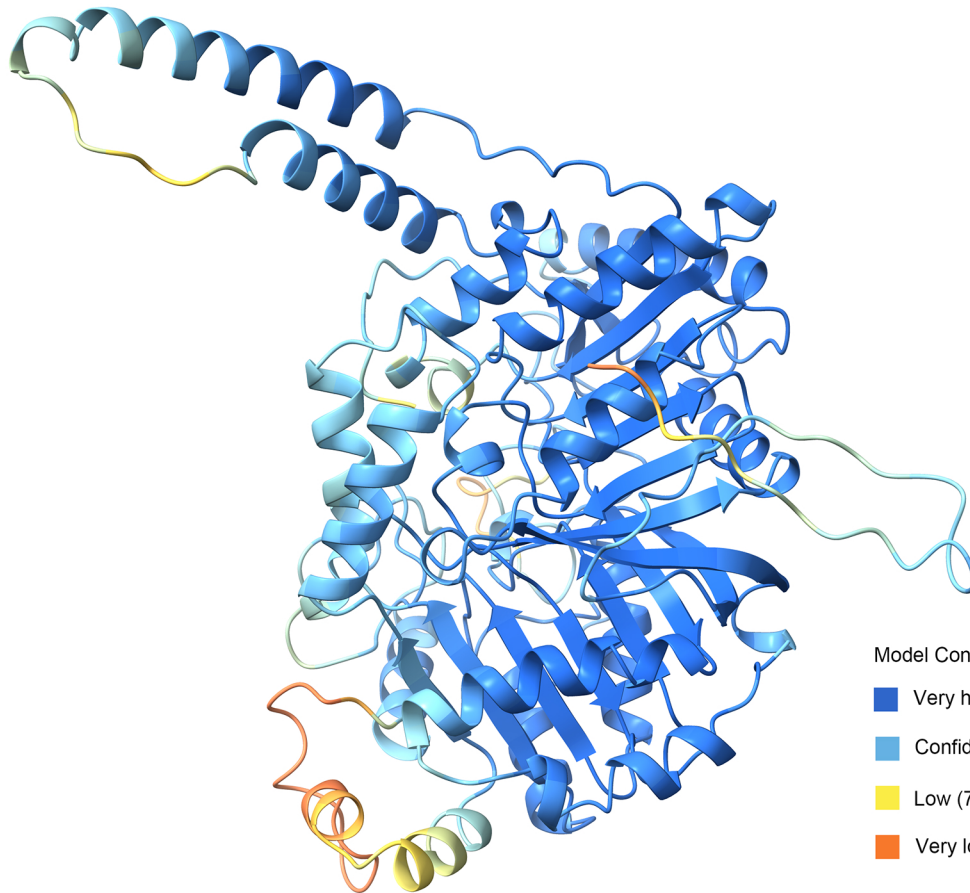

Model Confidence:

- Very high (pLDDT>90)
- Confident (90>pLDDT>70)
- Low (70>pLDDT>50)
- Very low (pLDDT<50)

Supplement: Supplementary file 3 [file Data_Sheet_3.PDF]
